# Comparisons of four cognitive-frailty measures in predicting dementia and disability

**DOI:** 10.1186/s12877-025-05874-0

**Published:** 2025-04-10

**Authors:** Jui-Yuan Chung, Hei-Fen Hwang, Lalu Suprawesta, Mau-Roung Lin

**Affiliations:** 1 Institute of Injury Prevention and Control, College of Public Health, Taipei Medical University, 250 Wu-Hsing Street, Taipei, 11031 Taiwan, ROC; 2 Department of Emergency Medicine, Cathay General Hospital, Taipei, Taiwan, ROC; 3 School of Medicine, National Tsing Hua University, Hsinchu, Taiwan, ROC; 4 Department of Nursing, National Taipei University of Nursing and Health Sciences, Taipei, Taiwan, ROC; 5 Department of Sport and Health Education, Faculty of Sport Science and Public Health, Universitas Pendidikan Mandalika, Mataram, West Nusa Tenggara Indonesia; 6 Programs in Medical Neuroscience, College of Medical Science and Technology, Taipei Medical University, Taipei, Taiwan, ROC

**Keywords:** Cognitive decline, Cognitive frailty, Frailty, Older adults, Prediction

## Abstract

**Background:**

Several cognitive-frailty (CF) measurements, such as traditional CF, the CF phenotype, physio-cognitive decline syndrome (PCDS), and motoric cognitive risk syndrome (MCRS) have been developed but their predictive abilities for incident dementia and incident disability are seldom compared. We conducted a 2-year prospective study to compare the associations of traditional CF, the CF phenotype, PCDS, and MCRS with incident dementia and incident disability.

**Methods:**

In total, 755 individuals aged 65 years or older, without preexisting dementia or disability, participated in the baseline assessment and were subsequently monitored over a 2-year period. Data on cognitive and frailty components of traditional CF, the CF phenotype, PCDS, and MCRS, were collected. The logistic regression model was used to investigate independent associations of each CF measure with incident dementia and incident disability.

**Results:**

In total, 505 participants completed the two annual follow-ups. After adjusting for other CF measures, age, and sex, incident dementia was significantly associated with PCDS (odds ratio [OR] = 2.54; 95% confidence interval [CI], 1.25 ~ 5.19) but was not significantly associated with traditional CF, the CF phenotype, or MCRS, and incident disability was significantly associated with the CF phenotype (OR = 2.90; 95% CI, 1.59 ~ 5.30) but was not significantly associated with traditional CF, PCDS, or MCRS. After adjusting for other CF measures, age, sex, educational level, and other variables, incident dementia was not independently associated with any CF measure, while the association of incident disability with the CF phenotype remained significant (OR = 2.72; 95% CI, 1.45 ~ 5.11).

**Conclusions:**

The CF phenotype, MCRS, and PCDS can possibly identify a higher number of CF cases than can the traditional CF measure. While the CF phenotype was a significant predictor of incident disability, all four CF measures lacked an independent association with incident dementia over a 2-year period. Future studies with a longer study period are needed to validate our results.

**Supplementary Information:**

The online version contains supplementary material available at 10.1186/s12877-025-05874-0.

## Background

Cognitive frailty (CF) is a syndrome that coexists with physical frailty and mild cognitive impairment (MCI) in the absence of dementia and other neurological disorders; it also is a precursor of neurodegenerative processes under different circumstances [1]. Approximately 70% of frail older adults also exhibit cognitive impairment [2], while half of cognitively impaired older adults also meet the Fried’s frailty criteria [3].

Cognitive frailty has been reported to increase the risks of adverse health outcomes, such as all-cause mortality, functional disability, dementia, poor quality of life, and suicidal ideation [2, 4–10]. Specifically, older adults with CF, relative to those with either frailty or cognitive impairment alone, face an elevated risk of developing dementia and limitations of activities of daily living (ADLs) [8–10]. To prevent dementia and disability in older adults, an effective strategy is to implement intervention programs targeting those diagnosed with CF [11, 12].

As established by the International Academy on Nutrition and Aging and the International Association of Gerontology and Geriatrics, this traditional definition characterizes CF as the concurrent presence of physical frailty and cognitive impairment [1]. However, the prevalence of CF based on this operational definition was very low, ranging 1% to 5% [2, 5, 9, 13, 14]. This low prevalence has limited clinical applicability for effective identification and intervention of CF cases [15]. On the other hand, a modified definition, CF phenotype, has broadened the traditional CF spectrum by combining pre-frailty or frailty with pre-MCI (subjective cognitive decline, SCD) as reversible cognitive frailty (RCF) and combining pre-frailty or frailty with MCI as potentially RCF (PRCF) [16]. Another two operational definitions of CF have also been formulated to provide a wider range of CF cases. The physio-cognitive decline syndrome (PCDS) is characterized by the presence of muscle weakness and/or gait slowness, accompanied by cognitive decline exceeding 1.5 standard deviations below age- and education-matched norms in any cognitive domain (cognitive impairment no dementia, CIND) [17–19]. The motoric cognitive risk syndrome (MCRS) is defined as the coexistence of a slow gait and SCD [20, 21], in which a slow gait, as a component of frailty criteria, is known to be associated with the development of cognitive and physical declines [22, 23]. Relatively to traditional CF, the CF phenotype, PCDS, and MCRS measures might identify different numbers of CF cases for clinical interventions and might have distinctive predictive capacities for incident dementia and incident disability.

This 2-year prospective study was conducted to examine differences in identifying numbers of CF cases and predicting incident dementia and incident disability among the four CF measures of the traditional CF, CF phenotype, MCRS, and PCDS.

## Methods

### Study participants and settings

**Fig. 1:**
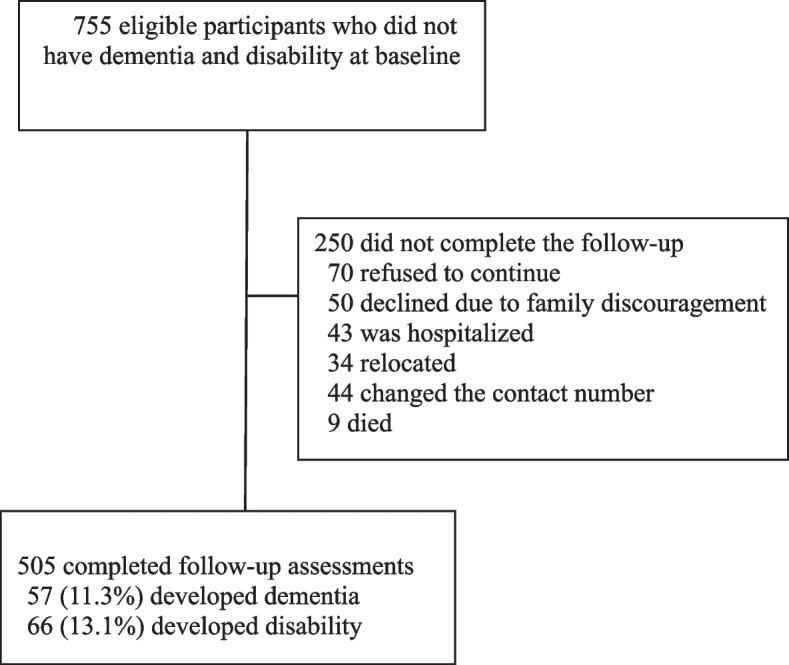
Flow diagram of study participants for incident dementia and incident disability developed during a 2-year study period

This study employed a prospective cohort design and recruited participants from outpatient clinics of Taipei Medical University Hospital over a period of August 2017 to December 2018 in Taipei, Taiwan. Inclusion criteria were individuals aged 65 years or older, capable of independent ambulation, and without recurrent or persistent care facility requirements. Individuals were excluded if they had communication difficulties, were unable to perform basic ADLs, had major health issues such as advanced cancer, major cardiopulmonary disease, or dementia, or did not participate in the follow-up evaluation due to family issues, transportation difficulties, weather conditions or poor health. The Institutional Review Board of Taipei Medical University approved the research protocol, and written informed consent was obtained from all participants. A flow diagram of study participants at the baseline and two annual follow-up assessments is illustrated in Fig. 1.

### Data collection

Information was gathered through individual interviews and performance assessments. At the baseline assessment, four CF measures (traditional CF, CF phenotype, MCRS, and PCDS) and covariates (sociodemographics, lifestyle factors, preexisting medical conditions, prescribed medications, balance assessments, depressive symptoms, cognitive measurements, and gait characteristics) were collected.

### CF measures

CF is defined as a heterogeneous clinical syndrome combining cognitive impairment and pre-frailty or frailty and is excluded if there is dementia resulting from Alzheimer’s disease (AD) or other conditions [15, 16]. Traditional CF is defined as the simultaneous presence of physical frailty and MCI, excluding Alzheimer’s disease and other types of dementia. The CF phenotype encompasses two subtypes: RCF, characterized by the combination of pre-frailty/frailty and SCD, and PRCF, characterized by the combination of pre-frailty/frailty and MCI. PCDS is indicated by the simultaneous presence of mobility impairment without disability (slowness or/and weakness) and CIND. CIND in this study was determined by cognitive performance decreasing by ≥ 1.5 standard deviations below the mean in all cognitive domains assessed using the Mattis Dementia Rating Scale (MDRS) [17]. MCRS is defined as the coexistence of slowness and SCD [20]. Individuals who had none of those conditions described in the four CF measures were indicated as having no CF [16].

In this study, cognitive impairment was determined by the MDRS, which contains five domains of attention, construction, initiation/perseveration, conceptualization, and memory. The total score of the MDRS ranges 0 ~ 144 points [24, 25]. In this study, MDRS scores of > 131, 126 ~ 131, and < 126 were used to indicate no dementia, MCI, and dementia, respectively [26]. Furthermore, the SCD status was indicated by a positive response to a question ('Do you feel that you have more problems with thinking and memory than most?'), with no evidence of objective cognitive impairment (MDRS score of > 131). On the other hand, the frailty component of the CF measures was assessed by five components of the frailty phenotype [27]: (1) unintentional weight loss, defined as a reduction in body weight exceeding 3 kg or 5% of the total body weight within the past year; (2) low grip strength, measured in kilograms of isometric force using a handgrip dynamometer, with the threshold for low grip strength set to ≤ 29 kg for males and ≤ 17 kg for females; (3) self-reported exhaustion, identified by a positive response to the question,'I felt that everything I did was an effort’; (4) slowness, characterized as having a gait velocity of < 0.8 m/s during normal walking; and (5) low physical activity, indicated by engaging in vigorous-intensity activity for fewer than 3 days per week, with each session lasting at least 20 min, or participating in moderate-intensity activity or walking for fewer than 5 days per week, with each session lasting at least 30 min. Physical activities were evaluated using the International Physical Activity Questionnaire-Short Form [28]. Overall, frailty was identified when three or more of these five components were present, while pre-frailty was determined by the presence of one or two components, and non-frailty was confirmed in the absence of all five components.

### Covariates

In this study, sociodemographics and lifestyle factors consisted of age, gender, body-mass index (BMI), educational attainment, monthly household income, regular exercise habits, current smoking status, and alcohol consumption. The BMI was classified into four categories: underweight (< 18.5 kg/m^2^), normal weight (18.5 ~ 22.9 kg/m^2^), overweight (23 ~ 24.9 kg/m^2^), and obesity (≥ 25 kg/m^2^) [29]. Comorbidities were assessed through 12 chronic diseases of hypertension, diabetes, heart disease, malignant tumors, respiratory tract diseases, arthritis or rheumatism, gastric ulcers, liver diseases, cataracts, kidney diseases, gout, and spinal spurs. The use of medications for these chronic diseases was documented. The Tinetti balance assesses a wide spectrum of 13 maneuvers in everyday activities, including both static and dynamic balance skills. Scores on the Tinetti balance range 0 to 24, with higher scores indicating better balance control [30]. The 15-item Geriatric Depression Scale (GDS) was employed to assess depressive symptoms, with a score exceeding 5 indicating the presence of depression [31]. Using a 6-m GAITRite electronic walkway (CIR Systems, Franklin, NJ, USA), gait velocity (cm/s) and cadence (steps/min) were assessed by averaging the results of two trials of usual walking pace over the walkway [32].

### Follow-up assessments of dementia and disability

After the baseline assessment, the cognitive status and ADLs were monitored at annual follow-ups to ascertain incident dementia and incident disability. In this study, incident dementia was indicated by an MDRS score of < 126 [33]. ADLs were assessed by the Older Adults Resources and Services (OARS) ADL scale, which contains seven basic ADLs and seven instrumental ADLs [34], and each of the 14 ADL items was assigned a score of 2 (inability to perform the activity), 1 (the need for some assistance), or 0 points (no need of assistance). Incident disability was indicated by impairment on any ADL item.

### Statistical analysis

CF measures and covariates at the baseline were compared between participants with incident dementia and those without and between participants with incident disability and those without. Continuous variables were assessed using an analysis of variance (ANOVA) test and Student's *t*-test, while categorical variables were evaluated through Pearson's Chi-squared test. To evaluate the presence of any selection bias in the study, baseline characteristics of participants who completed the follow-ups were compared to those who did not complete them.

A multivariable binary logistic regression model was used to investigate independent associations of traditional CF, the CF phenotype, MCRS, and PCDS with incident dementia and incident disability after adjusting for covariates, for which adjusted odds ratios (ORs) and 95% confidence intervals (CIs) are presented. In the beginning of the logistic regression analysis, associations of the four CF measures with incident dementia or incident disability were examined. Then, age and sex were adjusted due to their biological and clinical significance. Finally, in addition to age and sex, potential confounders were also adjusted for associations of the four CF measures with incident dementia or incident disability. The Statistical Package for the Social Sciences (SPSS) vers. 25.0 for Windows (IBM, Armonk, NY, USA) was used for all statistical analyses.

## Results

In total, 755 older adults who were not suffering from dementia and a disability participated in the baseline assessment, among which 505 completed two annual follow-ups. Compared to participants who completed the two follow-ups, those who did not complete them were significantly more likely to have the CF phenotype (36.8% vs. 28.5%), the lowest monthly household income (54.4% vs. 47.3%), no alcohol consumption (92.0% vs. 86.5%), and lower balance scores (the mean: 23.0 vs. 23.3 points) (Supplementary Table 1).

Of those 505 participants, 57 (11.3%) developed dementia while 66 (13.1%) developed disability over the 2-year study period. Distributions of the four CF measures with regard to incident dementia and incident disability are presented in Table 1. Compared to the non-dementia group, participants with incident dementia exhibited a significantly higher proportion of traditional CF (14.0% vs. 6.9%), the CF phenotype (45.6% vs. 26.3%), MCRS (22.8% vs. 8.5%), and PCDS (38.6% vs. 15.2%). Compared to participants without disability, those with disability showed a significantly higher proportion of traditional CF (19.7% vs. 5.9%), the CF phenotype (54.5% vs. 25.6%), and MCRS (24.2% vs. 8.0%).

**Table 1:** Distributions of four cognitive-frailty (CF) measures of traditional CF, CF phenotype, motoric cognitive risk syndrome (MCRS), and physio-cognitive decline syndrome (PCDS) with regard to incident dementia and incident disability during a 2-year study period

Characteristic	All (*N* = 505) *n* (%)	Dementia (*N* = 57) *n* (%)	No dementia (*N* = 448) *n* (%)	Disability (*N* = 66) *n* (%)	No disability (*N* = 439) *n* (%)
Traditional CF					
No CF	466 (92.3)	49 (86.0)	417 (93.1)	53 (80.3)	413 (94.1)*
CF	39 (7.7)	8 (14.0)	31 (6.9)	13 (19.7)	26 (5.9)
CF phenotype					
No CF phenotype	361 (71.5)	31 (54.4)	330 (73.7)*	30 (45.5)	331 (75.4)*
CF phenotype	144 (28.5)	26 (45.6)	118 (26.3)	36 (54.5)	108 (24.6)
MCRS					
No MCRS	454 (89.9)	44 (77.2)	410 (91.5)*	50 (75.8)	404 (92.0)*
MCRS	51 (10.1)	13 (22.8)	38 (8.5)	16 (24.2)	35 (8.0)
PCDS					
No PCDS	415 (82.2)	35 (61.4)	380 (84.8)*	50 (75.8)	365 (83.1)
PCDS	90 (17.8)	22 (38.6)	68 (15.2)	16 (24.2)	74 (16.9)

^*^ *p* < 0.05

Distributions of baseline characteristics with regard to incident dementia and incident disability are presented in Table 2. Compared to the non-dementia group, participants with incident dementia tended to be significantly older (mean age: 74.2 vs. 70.3 years) and female (45.6% vs. 68.3%), have a higher proportion of elementary school attainment or below (42.1% vs. 8.7%), a low monthly household income (59.6% vs. 45.8%), current smoking (10.5% vs. 3.3%), and depression (22.8% vs. 9.4%), and achieve lower scores on the Tinetti balance (means of 22.6 vs. 23.4 points), MDRS (means of 132.5 vs. 137.5 points), gait velocity (means of 103.4 vs. 113.7 cm/s), and gait cadence (means of 105.7 vs. 111.6 steps/min). Furthermore, compared to the non-disability group, participants with disability significantly tended to be older (mean ages of 72.3 vs. 70.5 years) and underweight (15.2% vs. 4.8%), have a higher proportion of low monthly household income (60.6% vs. 45.3%), physical inactivity (31.8% vs. 15.9%), ≥ 4 comorbidities (37.9% vs. 15.5%), and depression (18.2% vs. 9.8%), and achieve lower scores on the Tinetti balance (means of 22.3 vs. 23.4 points), gait velocity (means of 99.9 vs. 114.5 cm/s), and gait cadence (means of 106.9 vs. 111.5 steps/min).

**Table 2:** Distributions of baseline characteristics with regard to incident dementia and incident disability during a 2-year study period

Characteristic	All (*N* = 505) *n* (%) or mean ± SD	Dementia (*N* = 57) *n* (%) or mean ± SD	No dementia (*N* = 448) *n* (%) or mean ± SD	Disability (*N* = 66) *n* (%) or mean ± SD	No disability (*N* = 439) *n* (%) or mean ± SD
Age (years)	70.7 ± 5.0	74.2 ± 5.9	70.3 ± 4.7*	72.3 ± 5.7	70.5 ± 4.8*
Sex					
Men	173 (34.3)	31 (54.4)	142 (31.7)*	22 (33.3)	151 (34.4)
Women	332 (65.7)	26 (45.6)	306 (68.3)	44 (66.7)	288 (65.6)
Educational level					
College or above	268 (53.1)	13 (22.8)	255 (56.9)*	30 (45.5)	238 (54.2)
Senior and junior high	174 (34.5)	20 (35.1)	154 (34.4)	25 (37.9)	149 (33.9)
Elementary or below	63 (12.5)	24 (42.1)	39 (8.7)	11 (16.7)	52 (11.8)
Monthly household income (NTD)					
Low (< 49,999)	239 (47.3)	34 (59.6)	205 (45.8)*	40 (60.6)	199 (45.3)*
Middle (50,000 ~ 99,999)	186 (36.8)	20 (35.1)	166 (37.1)	24 (36.4)	162 (36.9)
High (≥ 100,000)	80 (16.2)	3 (5.3)	77 (17.7)	2 (3.0)	78 (17.8)
Body-mass index					
Underweight	31 (6.1)	5 (8.8)	26 (5.8)	10 (15.2)	21 (4.8)*
Normal weight	131 (25.9)	11 (19.3)	120 (26.8)	15 (22.7)	116 (26.4)
Overweight	144 (28.5)	18 (31.6)	126 (28.1)	17 (25.8)	127 (28.9)
Obesity	199 (39.4)	23 (40.4)	176 (39.3)	24 (36.4)	175 (39.9)
Regular exercise (≥ 3 times per week)					
No	91 (18.0)	10 (17.5)	81 (18.1)	21 (31.8)	70 (15.9)*
Yes	414 (82.0)	47 (82.5)	367 (81.9)	45 (68.2)	369 (84.1)
Current smoking					
No	484 (95.8)	51 (89.5)	433 (96.7)*	64 (97.0)	420 (95.7)
Yes	21 (4.2)	6 (10.5)	15 (3.3)	2 (3.0)	19 (4.3)
Alcohol consumption					
No	437 (86.5)	49 (86.0)	388 (86.6)	58 (87.9)	379 (86.3)
Yes	68 (13.5)	8 (14.0)	60 (13.4)	8 (12.1)	60 (13.7)
Number of comorbidities					
0 or 1	185 (36.6)	16 (28.1)	169 (37.7)	13 (19.7)	172 (39.2)*
2 or 3	227 (45.0)	28 (49.1)	199 (44.4)	28 (42.4)	199 (45.3)
≥ 4	93 (18.4)	13 (22.8)	80 (17.9)	25 (37.9)	68 (15.5)
Number of medications					
0 or 1	110 (21.8)	7 (12.3)	103 (23.0)	9 (13.6)	101 (23.0)*
2 or 3	248 (49.1)	29 (50.9)	219 (48.9)	28 (42.4)	220 (50.1)
≥ 4	147 (29.1)	21 (36.8)	126 (28.1)	29 (43.9)	118 (26.9)
Tinetti balance (0 ~ 24)	23.3 ± 1.3	22.6 ± 1.7	23.4 ± 1.3*	22.3 ± 2.2	23.4 ± 1.1*
GDS score					
≤ 5	450 (89.1)	44 (77.2)	406 (90.6)*	54 (81.8)	396 (90.2)*
> 5	55 (10.9)	13 (22.8)	42 (9.4)	12 (18.2)	43 (9.8)
MDRS score	136.9 ± 4.6	132.5 ± 4.0	137.5 ± 4.4*	135.7 ± 5.4	137.1 ± 4.5
Gait characteristic					
Velocity (cm/s)	112.6 ± 23.3	103.4 ± 26.4	113.7 ± 22.6*	99.9 ± 23.6	114.5 ± 22.7*
Cadence (steps/min)	110.9 ± 11.6	105.7 ± 13.4	111.6 ± 11.2*	106.9 ± 13.4	111.5 ± 11.2*

*GDS* Geriatric Depression Scale, *MDRS* Mattis Dementia Rating Scale, *NTD* new Taiwan dollar (the average exchange rate in 2023 was US$1.00≈NTD31.41, *SD* standard deviation

^*^p < 0.05

Distributions of baseline characteristics with regard to the four CF measures of traditional CF, the CF phenotype, MCRS, and PCDS are presented in Table 3. Among the four measures, significant differences were found in most of the baseline characteristics, including age, sex, educational level, monthly household income, BMI, regular exercise, the number of comorbidities, the number of medications, Tinetti balance, depression, gait velocity, and gait cadence, while significant differences in current smoking and alcohol drinking were also found.

**Table 3:** Distributions of baseline characteristics with regard to traditional cognitive-frailty (CF), CF phenotype, motoric cognitive risk syndrome (MCRS) and physio-cognitive decline syndrome (PCDS) during a 2-year study period

Characteristics	All (*N* = 505) mean ± SD or *n* (%)	Traditional CF (N = 39) mean ± SD or *n* (%)	CF phenotype (*N* = 144) mean ± SD or *n* (%)	MCRS (*N* = 51) mean ± SD or *n* (%)	PCDS (*N* = 90) mean ± SD or *n* (%)
Age (years)	70.7 ± 5.0	72.2 ± 5.3*	71.6 ± 5.5*	74.6 ± 5.7*	73.5 ± 5.4*
Sex					
Men	173 (34.3)	8 (20.5)	31 (22.9)*	13 (25.5)	24 (26.7)
Women	332 (65.7)	31 (79.5)	111 (77.1)	38 (74.5)	66 (73.3)
Educational level					
College or above	268 (53.1)	15 (38.5)	55 (38.2)*	21 (41.2)	23 (25.6)*
Senior and junior high	174 (34.5)	19 (48.7)	62 (43.1)	22 (43.1)	34 (37.8)
Elementary or below	63 (12.5)	5 (7.9)	27 (18.8)	8 (15.7)	33 (36.7)
Monthly household income (NTD)					
Low (< 49,999)	239 (47.3)	19 (48.7)	85 (59.0)*	27 (52.9)	58 (64.4)*
Middle (50,000 ~ 99,999)	186 (36.8)	16 (41.0)	42 (29.2)	18 (35.3)	24 (26.7)
High (≥ 100,000)	80 (16.2)	4 (10.3)	17 (11.8)	6 (11.8)	8 (8.9)
Body-mass index					
Underweight	31 (6.1)	2 (5.1)	8 (5.6)	3 (5.9)	4 (4.4)*
Normal weight	131 (25.9)	6 (15.4)	27 (18.8)	11 (21.6)	13 (14.4)
Overweight	144 (28.5)	15 (38.5)	43 (29.9)	16 (31.4)	29 (32.2)
Obesity	199 (39.4)	16 (41.0)	66 (45.8)	21 (41.2)	44 (48.9)
Regular exercise (≥ 3 times per week)					
No	91 (18.0)	24 (61.5)*	52 (36.1)*	20 (39.2)*	15 (16.7)
Yes	414 (82.0)	15 (38.5)	92 (63.9)	31 (60.8)	75 (83.3)
Current smoking					
No	484 (95.8)	36 (92.3)	136 (94.4)	48 (94.1)	88 (97.8)
Yes	21 (4.2)	3 (7.7)	8 (5.6)	3 (5.9)	2 (2.2)
Alcohol consumption					
No	437 (86.5)	35 (89.7)	131 (91.0)	47 (92.2)	83 (92.2)
Yes	68 (13.5)	4 (10.3)	13 (9.0)	4 (7.8)	7 (7.8)
Number of comorbidities					
0 or 1	185 (36.6)	7 (17.9)*	39 (27.1)*	11 (21.6)*	31 (34.4)
2 or 3	227 (45.0)	18 (46.2)	68 (47.2)	21 (41.2)	38 (42.2)
≥ 4	93 (18.4)	14 (35.9)	37 (25.7)	19 (37.3)	21 (23.3)
Number of medications					
0 or 1	110 (21.8)	4 (10.3)*	23 (16.0)*	5 (9.8)*	14 (15.6)
2 or 3	248 (49.1)	15 (38.5)	64 (44.4)	23 (45.1)	43 (47.8)
≥ 4	147 (29.1)	20 (51.3)	57 (39.6)	23 (45.1)	33 (36.7)
Tinetti balance (0 ~ 24)	23.3 ± 1.3	22.1 ± 2.0*	22.5 ± 2.0*	21.8 ± 2.2*	22.6 ± 2.0*
GDS score					
≤ 5	450 (89.1)	25 (64.1)*	114 (79.2)*	37 (72.5)*	76 (84.4)
> 5	55 (10.9)	14 (35.9)	30 (20.8)	14 (27.5)	14 (15.6)
MDRS score	136.9 ± 4.6	135.8 ± 3.8	134.7 ± 5.5*	135.0 ± 5.0*	132.8 ± 4.5*
Gait characteristic					
Velocity (cm/s)	112.6 ± 23.3	87.5 ± 25.1*	101.0 ± 24.3*	73.8 ± 13.4*	96.4 ± 26.3*
Cadence (steps/min)	110.9 ± 11.6	100.8 ± 14.4*	107.8 ± 13.5*	93.7 ± 12.6*	104.5 ± 13.3*

*GDS* Geriatric Depression Scale, *MDRS* Mattis Dementia Rating Scale, *NTD* new Taiwan dollar (the average exchange rate in 2023 was US$1.00≈NTD31.41, *SD* standard deviation

^*^p < 0.05

Table 4 presents results of the bivariable and multivariable logit regression analyses of CF measures for incident dementia. In the bivariable analysis, incident dementia was significantly associated with the CF phenotype (OR = 2.35; 95% CI, 1.34 ~ 4.12), MCRS (OR = 3.19; 95% CI, 1.58 ~ 6.43), and PCDS (OR = 3.51; 95% CI, 1.94 ~ 6.35), but was not significantly associated with traditional CF. In the multivariable analysis, after adjusting for the other three CF measures, incident dementia was significantly associated with PCDS (OR = 2.73; 95% CI, 1.41 ~ 5.28), but was not significantly associated with traditional CF, the CF phenotype, MCRS, or PCDS (model 1), and after adjusting for age and sex, the association of incident dementia and PCDS (OR = 2.54; 95% CI, 1.25 ~ 5.19) remained significant (model 2). After adjusting for age, sex, and education level, incident dementia was not significantly associated with any of the four CF measures.

**Table 4:** Results of the binary logit regression analysis of four cognitive-frailty (CF) measures with odd ratios (ORs) and 95% confidence intervals (CIs) for incident dementia

Characteristic	Bivariable analysis		Multivariable analysis					
	OR (95% CI)	*p* value	Model 1		Model 2^a^		Model 3^b^	
			OR (95% CI)	*p* value	OR (95% CI)	*p* value	OR (95% CI)	*p* value
Traditional CF (vs. no CF)	2.20 (0.96 ~ 5.05)	0.064	1.18 (0.42 ~ 3.31)	0.749	1.54 (0.51 ~ 4.66)	0.446	1.57 (0.51 ~ 4.80)	0.432
CF phenotype (vs. no CF phenotype)	2.35 (1.34 ~ 4.12)	0.003	1.63 (0.86 ~ 3.10)	0.138	1.89 (0.95 ~ 3.76)	0.069	1.56 (0.76 ~ 3.21)	0.222
MCRS (vs. no MCRS)	3.19 (1.58 ~ 6.43)	0.001	1.43 (0.56 ~ 3.67)	0.452	0.92 (0.33 ~ 2.58)	0.870	1.66 (0.57 ~ 4.85)	0.355

MCRS, motoric cognitive risk syndrome; PCDS, physio-cognitive decline syndrome

^a^Adjusted for age and sex

^b^Adjusted for age, sex, and educational level

Table 5 presents results of the bivariable and multivariable logit regression analyses of the four CF measures for incident disability. In the bivariable analysis, incident disability was significantly associated with traditional CF (OR = 3.90; 95% CI, 1.89 ~ 8.04), the CF phenotype (OR = 3.68; 95% CI, 2.16 ~ 6.25), and MCRS (OR = 3.69; 95% CI, 1.91 ~ 7.15) but was not significantly with PCDS. In the multivariable analysis, after adjusting for the other three CF measures, incident disability was significantly association with the CF phenotype (OR = 2.86; 95% CI, 1.58 ~ 5.17) but was not significantly with traditional CF, MCRS, or PCDS (model 4). After adjusting for age and sex, the CF phenotype (OR = 2.90; 95% CI, 1.59 ~ 5.30) was still significantly associated with incident disability (model 5), and even after adjusting for more covariates (BMI, monthly household income, and number of comorbidities), this significant association (OR = 2.72; 95% CI, 1.45 ~ 5.11) remained (model 6).

**Table 5:** Results of the binary logit regression analysis of four cognitive-frailty (CF) measures with odd ratios (ORs) and 95% confidence intervals (CIs) for incident disability

Characteristic	Bivariable analysis		Multivariable analysis					
	OR (95% CI)	*p* value	Model 4		Model 5^a^		Model 6^b^	
			OR (95% CI)	*p* value	OR (95% CI)	*p* value	OR (95% CI)	*p* value
Traditional CF (vs. no CF)	3.90 (1.89 ~ 8.04)	< 0.001	1.64 (0.68 ~ 3.96)	0.275	1.74 (0.71 ~ 4.24)	0.224	1.75 (0.68 ~ 4.51)	0.250
CF phenotype (vs. no CF phenotype)	3.68 (2.16 ~ 6.25)	< 0.001	2.86 (1.58 ~ 5.17)	0.001	2.90 (1.59 ~ 5.30)	0.001	2.72 (1.45 ~ 5.11)	0.002
MCRS (vs. no MCRS)	3.69 (1.91 ~ 7.15)	< 0.001	1.81 (0.75 ~ 4.36)	0.187	1.51 (0.61 ~ 3.73)	0.368	1.45 (0.57 ~ 3.70)	0.438
PCDS (vs. no PCDS)	1.58 (0.85 ~ 2.92)	0.146	0.91 (0.44 ~ 1.87)	0.793	0.80 (0.38 ~ 1.68)	0.556	0.86 (0.40 ~ 1.87)	0.701

*MCRS* motoric cognitive risk syndrome, *PCDS* physio-cognitive decline syndrome

^a^Adjusted for age and sex

^b^Adjusted for age, sex, body-mass index, monthly household income, and number of comorbidities

## Discussion

While a number of studies have explored the relationship between CF and the development of dementia and disability, there is a lack of research assessing differences and predictive capacities of alternative CF measures in the context of incident dementia and incident disability. Our study results indicated that among the four CF measures of traditional CF, the CF phenotype, MCRS, and PCDS, none was independently associated with incident dementia while only the CF phenotype was independently associated with incident disability.

Differences in identifying the number of cases among the four CF measures may have been partly attributable to variations in their composition of both physical and cognitive domains. Consistent with the low prevalence of CF reported by prior studies [15], the traditional CF in this study identified the smallest number of CF cases. Furthermore, similar to the result of a Chinese population-based study in which women accounted for 52.7% of CF cases [35] traditional CF exhibited a higher proportion of women (79.5%), compared to the other CF measures. The female-majority distribution in the traditional CF might be attributed to a higher prevalence of frailty usually occurring in women than men (e.g., 8.8% vs. 5.4% [36]), since women exhibit a greater susceptibility to rapid declines in resting energy expenditures for both skeletal muscle and total adipose tissues [37].

In this study, the CF phenotype, which expands the spectrum of the traditional CF by introducing RCF and PRCF, identified the largest number of CF cases among the four CF measures, yet it was still not independently associated with incident dementia. Since assessing SCD could be influenced by such factors as sex, stress, depressive symptoms, social culture, alcohol consumption, smoking, and educational level [38–43], the SCD diagnosis in RCF might lack specificity of CF identification which could reduce its statistical efficiency in examining the association between the CF phenotype and incident dementia. On the other hand, in this study, the CF phenotype exhibited a significant association with incident disability, aligning with findings from a Singaporean study that reported an increased risk of incident disability in individuals with RCF or PRCF in relation to those without CF [44]. It is known that the incidence rate of disability usually is notably higher in a frailty group compared to a cognitive impairment group (e.g., 45.4 vs. 21.3 per 1000 person-years [45]). Additionally, individuals indicated as being frail or in a pre-frail state often exhibited a greater likelihood of developing or worsening disability compared to the non-frail group (e.g., a relative risk of 1.66 [46]).

The PCDS was the only CF measure that could significantly predict incident dementia when the educational level was not controlled for. Several studies also identified the PCDS as a significant risk factor for dementia [12, 47]. While the PCDS assesses the cognitive domain through objective cognitive evaluation, targeting dysfunction in multiple cognitive domains, a study found that individuals with PCDS exhibited poor cognitive functions in working memory, verbal fluency, and processing speed [48]; however, the significant association of PCDS with incident dementia was no longer evident after adjusting for the educational level. It is known that higher education may provide a buffer against clinical expression of dementia, allowing individuals to better tolerate pathological changes associated with the condition [49]. In this study, a much higher proportion of older adults with an elementary education or below was identified by the PCDS, compared to the other three CF measures (36.7% vs. 7.9% ~ 18.8%); this observed difference in educational level could possibly be attributed to the disparity in the level of cognitive impairment assessed by the CF measures. For instance, the PCDS assesses cognitive impairment using a decline of at least 1.5 standard deviations below the mean across all five MDRS domains, thereby identifying poorer cognitive function, compared to the other three CF measures (means of MDRS scores: 132.7 vs. 134.7 and 135.8). As a consequence, older adults with the lowest level of education could have poorer cognitive performance, with more affected cognitive domains, accounting for the higher number of the participants in the PCDS group.

In this study, the prevalence of MCRS (10.1%) was consistent with previous findings [50], while it was notably lower than the prevalence of the CF phenotype or PCDS. The difference in prevalence rates may be attributed to the operational definition of MCRS. Unlike the traditional CF or CF phenotype that encompass five components of frailty criteria, the MCRS includes only one component (gait slowness) in assessing frailty. Relative to participants with other CF measures, those with MCRS were characterized by older ages and poorer gait velocities. It is well-known that there is an increasing prevalence of slow gait with advancing age [51], and gait velocity beyond the age of 70 years declines by approximately 15% in the usual walking pace and 20% in the fast walking pace [52].

There are some limitations to this study. First, as study participants were recruited from the outpatient department of a general hospital, they might not be fully representative of community-dwelling older individuals, and caution should be taken when generalizing the findings to healthier older adults with a lower prevalence of chronic diseases. Second, a substantial proportion of study participants did not complete the follow-up assessments and were more likely to be CF cases in this study. Due to differences existing in the operationalizations of the four CF measures, the loss to follow-up might have had differential impacts on the prevalence of case identification and the relationships of CF with incident dementia or incident disability across the four CF measures. Future studies might select a random sample of subjects lost to follow-up and intensively go after them to examine if the case identification and the relationships of CF measures with dementia/disability are affected by selecting factors such as household income, alcohol drinking, or balance ability. Third, the pathophysiology might differ among the four CF measures in relation to dementia, leading to variations in their predictive abilities of dementia and disability in this study. Fourth, normative data for MDRS may vary across different populations and thus the cognitive status of MCI or dementia defined by our MDRS cutoffs might not be valid, although, for example, the cutoff of 125 demonstrated both high sensitivity (85%) and high specificity (90%) for identifying dementia cases [33]. Fifth, Finally, it is important to note that the follow-up duration in this study was relatively short, and further studies with longer study periods are warranted to evaluate relationships of CF measures with dementia and disability. Specifically, the CF groups defined by the four CF measures may progress at different rates to dementia or disability. For example, due to a higher level of cognitive impairment, older people with PCDS, compared to those with RCF, could be more likely to progress towards dementia in a shorter period. Hence, the relationships of CF measures with dementia or disability at a longer study duration might considerably change.

## Conclusions

Among the four CF measures of traditional CF, the CF phenotype, MCRS, and PCDS, the latter three may identify a higher number of CF cases than the traditional CF, thus enhancing their applicability in case identification and clinical interventions. Furthermore, all four CF measures may lack an independent association with incident dementia in a 2-year study period, and the CF phenotype may be a significant predictor of incident disability. Future studies with longer study periods are needed to validate our findings.

## Supplementary Information

Supplementary Material 1.

## Data Availability

Data is provided within the manuscript or supplementary information files.

## Abbreviations

AD: Alzheimer's Disease
ADL: Activity of daily living
ANOVA: Analysis of Variance
BMI: Body-mass index
CF: Cognitive frailty
CIND: Cognitive impairment without dementia
GDS: Geriatric Depression Scale
MCI: Mild cognitive impairment
MCRS: Motoric cognitive risk syndrome
MDRS: Mattis Dementia Rating Scale
NTD: New Taiwan Dollar
OARS: Older Adults Resources and Services
PCDS: Physio-cognitive decline syndrome
PRCF: Potentially reversible cognitive frailty
RCF: Reversible cognitive frailty
SCD: Subjective cognitive decline
SD: Standard Deviation
SPSS: Statistical Package for the Social Sciences
