# Supplementary material for: Comparisons of four cognitive-frailty measures in predicting dementia and disability
Source: BMC Geriatr. 2025 Apr 10;25:245. doi: 10.1186/s12877-025-05874-0 (PMC11984219; doi:10.1186/s12877-025-05874-0)
Supplement: Supplementary file 1 — Supplementary Material 1. [file 12877_2025_5874_MOESM1_ESM.docx]

**Supplementary Table 1.** Characteristics of 755 participants who completed and those who did not complete the 2-year follow-ups of dementia and disability

| Characteristic | All  (*N*=755)  mean±SD or *n* (%) | Completed  (*N*=505)  mean±SD  or *n* (%) | Not completed  (*N*=250)  mean±SD  or *n* (%) | *p* value |
| --- | --- | --- | --- | --- |
| Traditional CF |  |  |  |  |
| No CF | 698 (92.5) | 466 (92.3) | 232 (92.8) | 0.798 |
| CF | 57 (7.5) | 39 (7.7) | 18 (7.2) |  |
| CF phenotype |  |  |  |  |
| No CF phenotype | 519 (68.7) | 361 (71.5) | 158 (63.2) | 0.021 |
| CF phenotype | 236 (31.3) | 144 (28.5) | 92 (36.8) |  |
| MCRS |  |  |  |  |
| No MCRS | 679 (89.9) | 454 (89.9) | 225 (90.0) | 0.966 |
| MCRS | 76 (10.1) | 51 (10.1) | 25 (10.0) |  |
| PCDS |  |  |  |  |
| No PCDS | 607 (80.4) | 415 (82.2) | 192 (76.8) | 0.080 |
| PCDS | 148 (19.6) | 90 (17.8) | 58 (23.2) |  |
| Age (years) | 70.7±5.0 | 70.7±5.0 | 70.7±5.1 | 0.931 |
| Sex |  |  |  |  |
| Male | 258 (34.2) | 173 (34.3) | 85 (34.0) | 0.944 |
| Female | 497 (65.8) | 332 (65.7) | 165 (66.0) |  |
| Educational level |  |  |  |  |
| College or above | 393(52.1) | 268 (53.1) | 125 (50.0) | 0.210 |
| Senior and junior high | 275 (36.4) | 174 (34.5) | 101 (40.4) |  |
| Elementary or below | 87 (11.5) | 63 (12.5) | 24 (9.6) |  |
| Monthly household income (NTD) | |  |  |  |
| Low (<49,999) | 375 (49.7) | 239 (47.3) | 136 (54.4) | 0.031 |
| Middle (50,000~99,999) | 254 (33.6) | 186 (36.7) | 68 (27.2) |  |
| High (≥100,000) | 126 (16.7) | 80 (15.8) | 46 (18.4) |  |
| Body mass index |  |  |  |  |
| Underweight | 45 (6.0) | 31 (6.1) | 14 (5.6) | 0.204 |
| Normal weight | 214 (28.3) | 131 (25.9) | 83 (33.2) |  |
| Overweight | 212 (28.1) | 144 (28.5) | 68 (27.2) |  |
| Obesity | 284 (37.6) | 199 (39.4) | 85 (34.0) |  |
| Regular exercise (≥3 times per week) | |  |  |  |
| No | 141 (18.7) | 91 (18.0) | 50 (20.0) | 0.511 |
| Yes | 614 (81.3) | 414 (82.0) | 200 (80.0) |  |
| Current smoking |  |  |  |  |
| No | 722 (95.6) | 484 (95.8) | 238 (95.2) | 0.685 |
| Yes | 33 (4.4) | 21 (4.2) | 12 (4.8) |  |
| Alcohol consumption |  |  |  |  |
| No | 667 (88.3) | 437 (86.5) | 230 (92.0) | 0.028 |
| Yes | 88 (11.7) | 68 (13.5) | 20 (8.0) |  |
| Number of comorbidities |  |  |  |  |
| 0 or 1 | 276 (36.6) | 185 (36.6) | 91 (36.4) | 0.548 |
| 2 or 3 | 332 (44.0) | 227 (45.0) | 105 (42.0) |  |
| ≥4 | 147 (19.5) | 93 (18.4) | 54 (21.6) |  |
| Number of medications |  |  |  |  |
| 0 or 1 | 160 (21.2) | 110 (21.8) | 50 (20.0) | 0.735 |
| 2 or 3 | 369 (48.9) | 248 (49.1) | 121 (48.4) |  |
| ≥4 | 226 (29.9) | 147 (29.1) | 79 (33.1) |  |
| Tinetti balance (0~24) | 23.2±1.4 | 23.3±1.3 | 23.0±1.6 | 0.006 |
| GDS score |  |  |  |  |
| ≤5 | 662 (87.7) | 450 (89.1) | 212 (84.8) | 0.090 |
| >5 | 93 (12.3) | 55 (10.9) | 38 (15.2) |  |
| MDRS score | 136.7±4.5 | 136.9±4.6 | 136.3±4.3 | 0.031 |
| Gait characteristic |  |  |  |  |
| Velocity (cm/s) | 113.1±22.3 | 112.6±23.3 | 114.1±20.0 | 0.447 |
| Cadence (steps/min) | 111.4±11.5 | 110.9±11.6 | 112.3±11.1 | 0.281 |

CF, cognitive frailty; GDS, Geriatric Depression Scale; MCRS, motoric cognitive risk syndrome; MDRS, Mattis Dementia Rating Scale; PCDS, physio-cognitive decline syndrome; NTD, new Taiwan dollar (the average exchange rate in 2023 was US$1.00≈NTD31.41; SD, standard deviation.
